# Extracellular Vesicles as Therapeutic Agents for Cardiac Fibrosis

**DOI:** 10.3389/fphys.2020.00479

**Published:** 2020-05-21

**Authors:** Russell G. Rogers, Alessandra Ciullo, Eduardo Marbán, Ahmed G. Ibrahim

**Affiliations:** Smidt Heart Institute, Cedars-Sinai Medical Center, Los Angeles, CA, United States

**Keywords:** bioengineering, cardiac fibrosis, extracellular vesicles, progenitor cells, next generation therapeutics

## Abstract

Heart disease remains an increasing major public health challenge in the United States and worldwide. A common end-organ feature in diseased hearts is myocardial fibrosis, which stiffens the heart and interferes with normal pump function, leading to pump failure. The development of cells for regenerative therapy has been met with many pitfalls on its path to clinical translation. Recognizing that regenerative cells secrete therapeutically bioactive vesicles has paved the way to circumvent many failures of cell therapy. In this review, we provide an overview of extracellular vesicles (EVs), with a focus on their utility as therapeutic agents for cardiac regeneration. We also highlight the engineering potential of EVs to enhance their therapeutic application.

## Introduction

Despite significant advances in science and medicine, heart disease remains an increasing public health concern, and is a leading cause of morbidity and mortality worldwide (Roth et al., 2017). In adult mammals, the default response to cardiac insults or disease is scar formation which lowers myocardial compliance, decreases ventricular filling, interferes with electrical coupling, and ultimately leads to depressed cardiac performance (Sharma and Kass, 2014). Conventional therapies such as β-blockers are beneficial in patients with myocardial fibrosis; however, they do not directly treat the underlying causes of fibrosis (Jameel and Zhang, 2009; Members et al., 2012). There is ample evidence for the efficacy of cardiac cell therapy to treat myocardial fibrosis in preclinical models (Zwetsloot et al., 2016) and, to a lesser extent, in patients (Nigro et al., 2018). However, cells are fragile, living entities which can be difficult to manufacture and to handle (Dodson and Levine, 2015).

In recent years, the emphasis has shifted away from cell therapy toward a cell-free therapeutic paradigm. Although extracellular vesicles (EVs) have long been known to be produced by eukaryotic cells, only recently were EVs implicated as mediators of the paracrine benefits of cell therapy. Extensive evidence now supports the concept that EVs are vital for the benefits of numerous therapeutic cells such as neural progenitors (Marzesco et al., 2005), mesenchymal stem cells (Lai et al., 2010), CD34+ cells (Sahoo et al., 2011), and cardiosphere-derived cells (CDCs; Ibrahim et al., 2014). Importantly, EVs offer the potential to overcome key limitations of cell therapy. For example, advantages may include product stability (Akers et al., 2016), immune tolerability (Gallet et al., 2017; Aminzadeh et al., 2018), flexibility of dosing (not limited by microvascular plugging or loss of transplanted cell viability; Ibrahim and Marbán, 2016), and the potential for engineering to enhance efficacy (Conlan et al., 2017). In this review, we summarize EV biology, cellular and molecular players in myocardial fibrosis, the utility of EVs as therapeutic agents, and conclude with the promise of engineered EVs as next-generation therapeutic candidates.

## Extracellular Vesicles Defined

Conserved through evolution, cellular release of EVs occurs in bacteria, fungi, plants, and animals (Barile et al., 2017). Based on studies of sheep reticulocytes, EV secretion was originally postulated to be a mechanism for elimination of cell waste including non-essential proteins (Johnstone et al., 1987). More recently, EVs have come to be viewed as key mediators of intercellular communication. EVs can deliver and exchange bioactive components from donor to recipient cells, regulating gene expression and altering cellular function (Stahl and Raposo, 2018). Several lines of evidence implicate EVs as important signaling mediators that carry proteins, lipids, and nucleic acids in physiological and pathophysiological conditions (Ibrahim et al., 2014; Ibrahim and Marbán, 2016).

### Classification of Extracellular Vesicles

**FIGURE 1:**
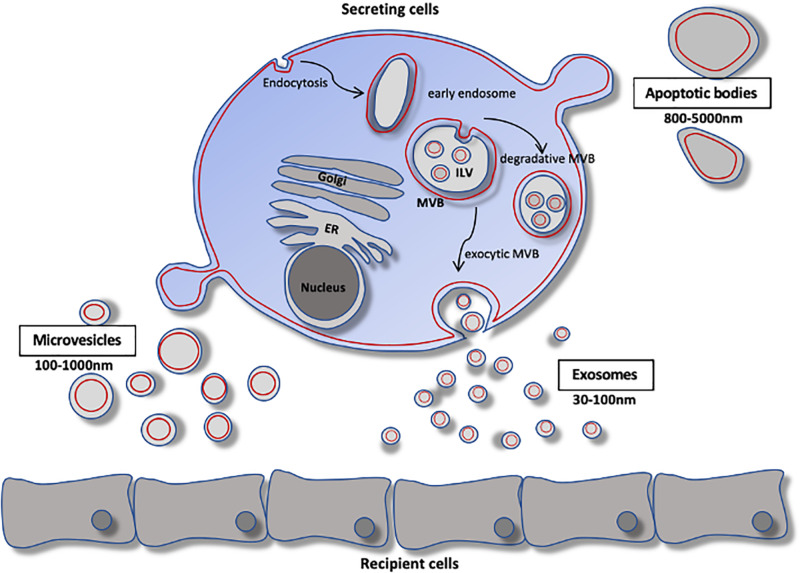
Extracellular vesicle biology. Schematic of extracellular vesicle biogenesis. Exosomes arise from the fusion of inward budding of multivesicular bodies. The resulting vesicles are either degraded by lysosomes or secreted as exsomes. Microvesicles arise by direct budding from the plasma membrane and enter the extracellular space. Apoptotic bodies arise from membrane protrusions fragmentation of an apoptotic cell. ER, endoplasmic reticulum; ILV, intraluminal vesicle; MVB, multivesicular body.

In the classical sense, the term EVs refers to all cell-secreted membrane vesicles. Based on their size, biogenesis, and secretory pathway, EVs can be broadly classified into three major classes: exosomes, microvesicles, and apoptotic bodies (Figure 1). The primary focus here will be on the first two classes of EVs.

*Exosomes* are secreted and taken up by all eukaryotic cells and have been found abundantly in nearly all biological fluids. Exosomes are lipid-bilayer vesicles of endosomal origin that arise from inward budding of multivesicular bodies, and range in size from 30 to ∼100 nm in diameter. Multivesicular bodies can fuse with the plasma membrane to release their contents into the extracellular space, or can be trafficked to lysosomes for degradation (Colombo et al., 2014). Exosomal cargo contains non-random assortments of protein, RNA, and lipids, differing substantially from the cytoplasmic contents of the parent cells, but nevertheless reflecting the parent cell type and its metabolic state. According to ExoCarta^1^, at least 9,769 proteins, 4,946 mRNAs, 2,838 miRNAs, and 1,116 lipids have been identified in exosomes from various cell types. Though the diversity of exosomal cargo is vast, exosomes share common molecular markers including tetraspanins (CD9, CD63, and CD81). However, diversity exists among the expression of these tetraspanins – although the biological significance is currently unclear (Bobrie et al., 2012).

In contrast to exosomes, *microvesicles* are generally larger and range in size (100–1000 nm in diameter). The mechanism of microvesicle biogenesis is not well understood; however, it is thought to require cytoskeletal components such as actin and microtubules (along with the respective molecular motors), and fusion machinery (SNAREs and tethering proteins) (Cai et al., 2007). Microvesicles arise by direct budding from the plasma membrane and enter the extracellular space (He et al., 2018). Accordingly, microvesicle contents closely resemble the composition of the cytosol of the parent cell (Mohammadi et al., 2019).

We next consider the biology of cardiac fibrosis, then return to EVs as therapeutic candidates to offset fibrosis. For a detailed review of cardiac regeneration and EV biology, the reader is referred to recently published reviews (Balbi et al., 2020; Tikhomirov et al., 2020).

## Origin of Cardiac Fibrosis

**FIGURE 2:**
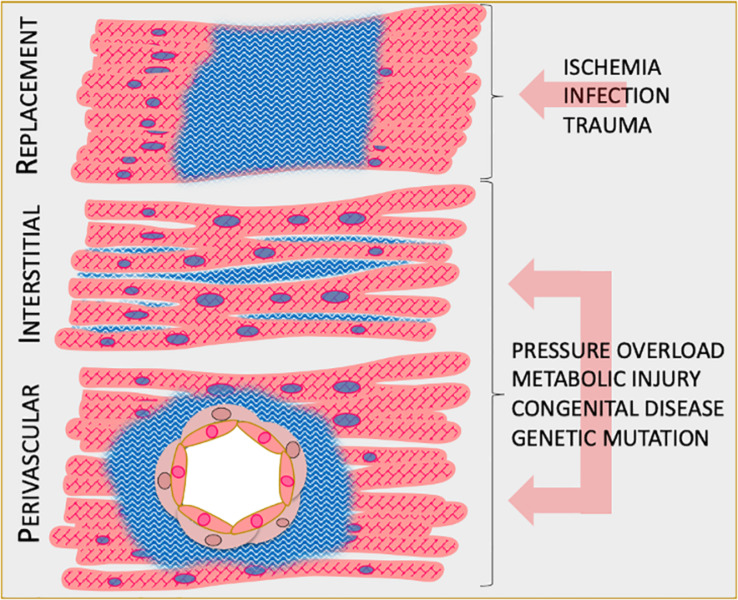
Manifestations of cardiac fibrosis. Schematic of the three main types of cardiac fibrosis. Replacement fibrosis develops to fill necrotic lesions within the myocardium as a result of ischemia, infection, or trauma. Interstitial fibrosis develops in the interstitial space of the myocardium as a result of non-ischemic cardiomyopathies. Perivascular fibrosis develops in the peri-adventitial space as a result of non-ischemic cardiomyopathies.

The deposition of collagen occurs in three forms: replacement, interstitial, and perivascular (Figure 2). As a result of cardiomyocyte loss, replacement fibrosis ensues to fill devoid space within the myocardium (Rogers and Otis, 2017). Dominating in acute myocardial infarction (MI), replacement fibrosis is critical to protecting the myocardium from rupture and dilative remodeling by preserving structural integrity and normalizing myocardial wall stress. Interstitial and perivascular fibrosis develop as excess collagen deposits in the myocardial interstitium and surrounding the peri-adventitia, respectively (Frangogiannis, 2019). The latter two are the unfortunate consequences of a number of insults, including myocardial inflammation, which lead to chronic heart disease. The extent of interstitial and perivascular fibrosis is closely associated with adverse clinical outcomes (Berk et al., 2007; Kong et al., 2014). As such, the development of therapeutic interventions to combat myocardial fibrosis remain a major focus of current research.

### Cellular and Molecular Players in Cardiac Fibrosis

The hallmark of any cardiac insult remains the universal activation of the innate and adaptive immune system. For example, following acute myocardial infarction, dead cardiomyocytes release DNA (Roers et al., 2016) and cellular proteins (Scaffidi et al., 2002; Panayi et al., 2004) into the extracellular space which serve as damage-associated molecular patterns (Rubartelli and Lotze, 2007). These signals are sensed by leukocytes (Tang et al., 2012) and activate nuclear factor kappa-light-chain-enhancer of activated B cells (NFκB)-mediated transcription of pro-inflammatory cytokines, chemokines, and other mediators of the inflammatory response (Legrand et al., 2019). Mounting evidence suggests infiltrating macrophages are key mediators of the pro-fibrotic response to injury (Frangogiannis, 2019). In animal models of pressure overload-induced heart failure, recruited M1 macrophages communicate with CD4^+^ T lymphocytes and perpetuate the pro-inflammatory wave leading to fibrosis, cardiac dysfunction, and heart failure (Nevers et al., 2015; Patel et al., 2018). Moreover, macrophages can activate resident stromal cells which contribute directly to collagen deposition. For example, in response to angiotensin-induced hypertrophic cardiomyopathy, macrophages stimulate cardiac fibroblasts to produce IL-6 leading to TGFβ1 production and subsequent development of cardiac fibrosis (Ma et al., 2012).

Perhaps the most widely known macrophage-secreted cytokine to drive fibrosis is TGFβ, which binds to its cognate receptors on cardiac tissue (TGFβR2, ALK, and others) and mobilizes Smad2 and Smad3 transcription factors to initiate myofibroblast differentiation (Khalil et al., 2017; Pardali et al., 2017). The principal source of myofibroblasts in the heart are resident fibroblasts, and to a lesser degree, endothelial cells. However, not all macrophages are created equal. M2c macrophages, for example, phagocytose dead cells and debris and secrete matrix metalloproteinases, which can reabsorb collagen deposits and contribute to remodeling of the extracellular matrix (Cheng et al., 2018; Krzyszczyk et al., 2018). The interplay among macrophages, fibroblasts, and endothelial cells is now understood to be a major driving force of myocardial fibrosis. Thus, in the context of anti-fibrogenesis, EVs can intervene by direct stimulation of pro-inflammatory M1 macrophages to differentiate into an M2-like phenotype (Silva et al., 2017). This step is important for the resolution of inflammation and subsequent anti-fibrotic actions. We will next discuss, in more detail, immunomodulatory actions of EVs.

## Extracellular Vesicles as Therapeutic Agents

**FIGURE 3:**
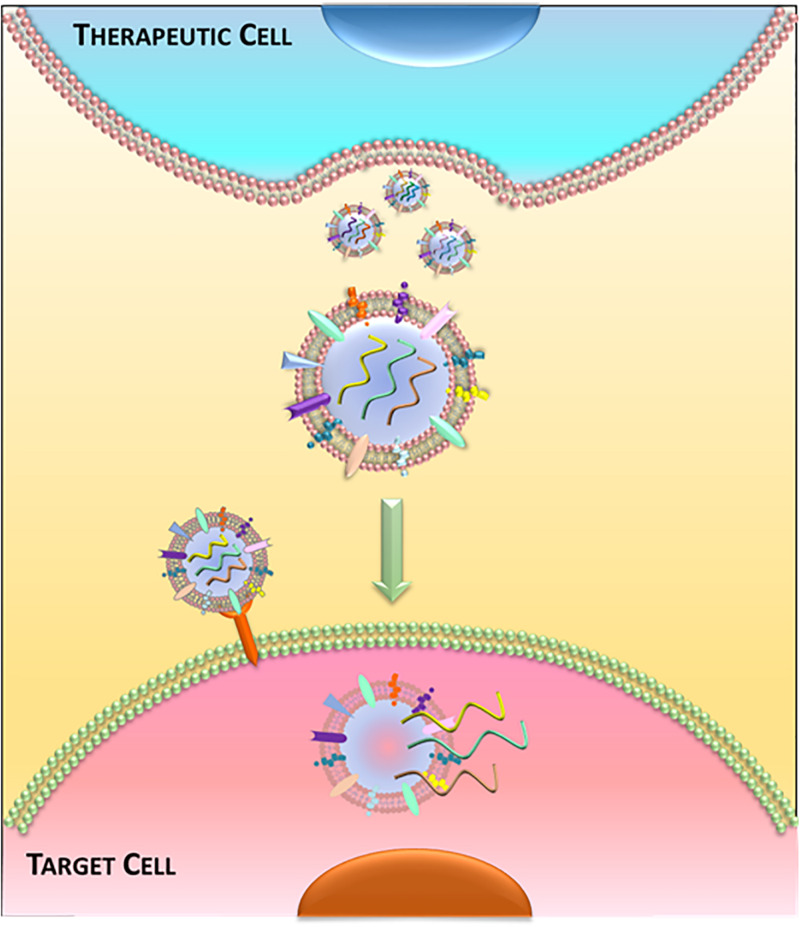
Extracellular vesicle-mediated communication. Schematic of release and uptake of extracellular vesicles. Therapeutic cells secrete bioactive extracellular vesicles, which are taken up by diseased cells to alter cell function.

**FIGURE 4:**
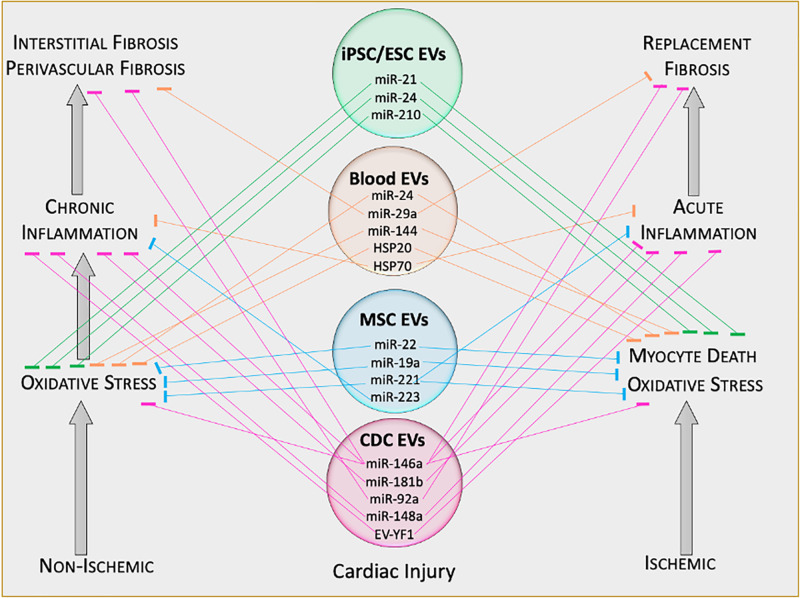
Therapeutic functions of extracellular vesicle cargo. Schematic of therapeutically active cargo found inside extracellular vesicles. Defined molecules exert specific biological functions in target tissues. EV, extracellular vesicle; ESC, embryonic stem cell; iPSC, induced pluripotent stem cell; miR, microRNA; EV-YF1, extracellular vesicle Y-RNA fragment.

Recently, EVs (and exosomes in particular) are attracting much interest – not only in physiological and pathological cell–cell communication, but also as a platform for therapeutic development (Sluijter et al., 2018). The recognition that progenitor cells secrete EVs that are bioactive ushered in the concept of EVs as cell-free therapeutic candidates (Figure 3). As the holy grail of regenerative medicine, restoring both cardiac structure and function are fundamental goals of any therapeutic candidate. Indeed, EVs harvested from cardiac stem/progenitor cells, mesenchymal stem/stromal cells (MSCs), embryonic stem cells (ESCs), induced pluripotent stem cells (iPSCs), and non-stem cell sources have demonstrated benefits in cardiac regeneration (Alibhai et al., 2018; Figure 4). By supplanting cell transplantation with administration of EVs, many concerns and limitations regarding safety and feasibility from cell therapy can be attenuated.

### Production of EVs for Therapeutic Applications

Therapeutic exosomes are of great potential interest, but it should be noted that there are no FDA-approved EV products. Nevertheless, some scholarship on the topic of isolating such exosomes has appeared (for example, Marbán, 2018b). Briefly, we start by culturing therapeutically potent cells *in vitro* in serum-free media (to exclude contaminating exosomes naturally found in serum), and allowing the cells to condition the media. The conditioning phase is variable and can be as brief as 24 h or as long as 15 days under normoxic or hypoxic conditions. Varying conditioning phase parameters can influence cargo loading into exosomes, which may impact their disease-modifying bioactivity. Conditioned media contains not only exosomes but also other EVs, proteins, and products of metabolism. To remove non-EV components, further processing by laboratory personnel typically involves ultrafiltration (i.e., by molecular weight exclusion). In order to separate exosomes from larger EVs such as microvesicles or apoptosomes, ultracentrifugation or size exclusion chromatography may be used (Baranyai et al., 2015). Finally, the purified product is aliquoted into dosage-defined units and packaged into vials for later use.

### Cardiosphere-Derived Cell EVs

Cardiosphere-derived cells (CDCs) are stromal cells of intrinsic cardiac origin (White et al., 2013), and are multipotent and clonogenic (but not self-renewing; Davis et al., 2009). CDCs uniformly express CD105 and are negative for CD45 and other hematogenous markers. Since the isolation of CDCs was first reported in 2007 (Smith et al., 2007), >200 papers have been published using this cell type from >55 independent labs worldwide. CDCs secrete EVs (CDC-EVs) which transfer payloads into target cells, inducing epigenomic, transcriptomic, and phenotypic changes that underlie the benefits of CDC therapy (Marbán, 2018b). Indeed, therapeutic bioactivity by CDC-EVs has been demonstrated in several animal studies such as acute MI (Ibrahim et al., 2014; De Couto et al., 2017; Gallet et al., 2017), non-ischemic cardiomyopathy (Aminzadeh et al., 2015), and Duchenne muscular dystrophy (DMD)-related cardiomyopathy (Aminzadeh et al., 2018; Rogers et al., 2019b). In preclinical studies, CDC-EVs induce cardiomyogenesis and angiogenesis, reduce fibrosis, modulate the immune response, and generally improve cardiac function (Marbán, 2018b). The mechanism of benefit appears to hinge on their cargo, particularly non-coding ribonucleic acids (ncRNA). Of the numerous RNA species, microRNAs (miRs) are the best-described class of small ncRNA.

Several CDC-EV associated miRs impact on inflammation and fibrosis including miR-146a (Barile et al., 2014; Ibrahim et al., 2014), miR-210 (Barile et al., 2014), miR-181b (De Couto et al., 2017), miR-148a (Aminzadeh et al., 2018), and miR-92a (Ibrahim et al., 2019). miR-146a targets multiple pathways active in cardiac disease including inflammation and fibrosis. Indeed, miR-146a regulates NFκB (Saba et al., 2014) and TGFβ signaling (Geraldo et al., 2012). Specifically, miR-146a inhibits Smad4-mediated myofibroblast differentiation to attenuate myocardial fibrosis (Liu et al., 2012). Delivery of a miR-146a mimic in infarcted mouse hearts reproduced some, but not all the benefits observed with CDC-EV treatment, suggesting cooperative effects of other CDC-EV cargo in the overall benefits. Moreover, it is unclear if other CDC-EV miRs share overlapping or synergistic bioactivity. For example, miR-181b blunts pro-inflammatory cytokine production by targeting protein kinase Cδ in a rat model of acute MI (De Couto et al., 2017), while miR-148a attenuates NFκB phosphorylation in the *mdx* mouse model of DMD (Aminzadeh et al., 2018; Rogers et al., 2019b). We have recently demonstrated CDC-EVs modulate the *mdx* mouse macrophage toward a pro-regenerative phenotype with prominent secretion of tissue inhibitor of matrixmetalloproteinase 2 (TIMP-2; Rogers et al., 2019a). We speculate that enhanced TIMP-2 secretion may contribute to the anti-fibrotic effect of CDC-EVs. In TIMP-2 null mice, MI led to greater ventricular dilation and infarct expansion (Kandalam et al., 2010). In contrast, TIMP-2 overexpression reduced ventricular dilation and infarct expansion post-MI (Ramani et al., 2011), and TIMP-2 inhibits human fibroblast activation at high concentrations (Ngu et al., 2014).

In addition to miRs, other less described ncRNAs, such as Y-RNAs, have been shown to influence the transcriptome in cardiac tissue. Y-RNAs are components of the Ro60 ribonucleoproteins and play a key role in DNA replication by interacting with chromatin and replication initiation proteins (Christov et al., 2006; Zhang et al., 2011). EV-YF1, a Y-RNA fragment found abundantly in CDC-EVs, skews macrophage polarization toward a pro-regenerative phenotype, which provides cardioprotection against ischemia/reperfusion injury in rats (Cambier et al., 2017). Moreover, in a mouse model of angiotensin-induced hypertrophic cardiomyopathy, EV-YF1 treatment attenuated myocardial hypertrophy, inflammation, and fibrosis, which were mediated by IL-10 (Cambier et al., 2018). While much work has focused on well-known ncRNAs such as miRs, it is clear that other RNA species contained within EVs are therapeutically bioactive. Further work will be required to better understand the role other lesser-known ncRNAs play in the mechanistic basis of EV-based therapeutics.

### Mesenchymal Stem Cell-Derived EVs

MSCs are multipotent stem/stromal cells found in loose connective tissue (e.g., areolar, reticular, and adipose), bone marrow, and lymph tissue (Bianco et al., 2008; Choi et al., 2019). While initial enthusiasm regarding MSCs originated from their capacity to differentiate into various cell types, it is now widely accepted that their engraftment and differentiation are negligible, and do not account for the therapeutic effects of MSC infusions (Iso et al., 2007; Leiker et al., 2008; Keating, 2012; Tokita et al., 2016). In congruency with CDCs (though a distinctly different cell type), MSCs mediate their disease-modifying bioactivity through the secretion of paracrine factors including EVs (MSC-EVs). Moreover, like CDC-EVs, MSC-EVs contain a plethora of RNA species, including miRs. For example, MSC-EVs from bone marrow-derived MSCs are enriched with miR-22, which confers anti-apoptotic and anti-fibrotic properties in a mouse model of acute MI (Feng et al., 2014). Further, cardioprotective miRs miR-19a and miR-221 are commonly found in MSC-EVs (Yu et al., 2013; Yu et al., 2015; Shi et al., 2018). Unlike MSCs, MSC-EVs are said to have no risk of tumorigenicity or extraosseous calcification, and a lower possibility of immune rejection following *in vivo* allogeneic administration (Stoltz et al., 2015). These features further support the notion of EVs as next-generation therapeutic candidates.

### Embryonic Stem Cell- and Induced Pluripotent Stem Cell-Derived EVs

Embryonic stem cells (ESCs) and induced pluripotent stem cells (iPSCs), like CDCs and MSCs, secrete bioactive EVs against cardiac injury and fibrosis, although they are traditionally believed to work by engraftment and differentiation rather than by paracrine mechanisms (Marbán, 2018a). ESC-EVs are enriched in the miR-290/295 cluster; one member, miR-294, promotes cardiomyocyte survival and attenuates fibrosis in MI (Khan et al., 2015). Similarly, iPSC-EVs have also demonstrated cardioprotective properties, mediated in part by miR-21 and miR-210 (Wang et al., 2015).

### Peripheral Blood-Derived EVs

Secreted mostly by platelets and the vascular endothelium, peripheral blood-derived EVs (PB-EVs) exhibit tissue-protective bioactivity. PB-EVs from healthy humans or rodents exert anti-oxidant (Vicencio et al., 2015), anti-apoptotic (Minghua et al., 2018), and anti-fibrotic (Yamaguchi et al., 2015) effects in animal models of MI. These therapeutic benefits appear to be, in part, mediated by miRs. The anti-fibrotic effects appear to be largely driven by miR-29, which has been validated to target col1a1, col1a2, col3a1, fbn1, and eln1 transcripts (Van Rooij et al., 2008). Moreover, PB-EVs were demonstrated to attenuate fibrosis and cardiac dysfunction in a streptozotocin-induced diabetic cardiomyopathy model, which was mediated, in part, by HSP20 (Wang et al., 2016).

## The Promise of Extracellular Vesicle Engineering for Next-Generation Therapeutics

The nature and the physiological state of the EV-secreting cell affects the tropism and therapeutic bioactivity of the produced vesicles (Wiklander et al., 2015; Lai et al., 2016). In the circulation, the balance between EV production and clearance reflects the steady-state level. Upon systemic delivery, the detection window of labeled EVs is typically very short (Yáñez-Mó et al., 2015). Generally speaking, EVs are distributed to many body tissues including the liver, bone, skin, muscle, spleen, kidney, and lung. EV-specific expression of selective adhesion molecules may influence biodistribution. For example, CD169-expressing macrophages in the spleen and lymph nodes capture B cell-derived EVs. In contrast, EV trafficking to the lymphoid system is dysregulated in CD169 knockout mice (Saunderson et al., 2014). Other insights into cell-specific EV uptake indicate the potential influence of saccharides. In the presence of D-mannose or D-glucosamine, EV uptake by dendritic cells was blunted, suggesting an EV uptake mechanism based on C-type lectin interaction (Hao et al., 2007). However, other studies have demonstrated sugars do not seem to play a significant role in EV-cell interaction and uptake (Escrevente et al., 2011), suggesting cell- or condition-specific difference in EV uptake mechanisms.

Given the identification of EV-derived biomolecules that mediate many of the therapeutic benefits associated with EV therapy, selectively loading EVs with defined biomolecules is one goal which can be achieved using various approaches reviewed elsewhere (Kenari et al., 2020). Briefly, a non-invasive method for loading therapeutic molecules into EVs is via co-incubation (Zhuang et al., 2011). This method allows hydrophobic biomolecules to enter the EV lumen through passive diffusion without disrupting the lipid membrane. Other methods include, but not limited to, electroporation or sonication (Tian et al., 2014; Kim et al., 2016). Although these methods have been successfully used to load exogenous biomolecules into EVs, they disrupt the lipid membrane and may result in EV damage or lysis. Further research will be required to determine if these are viable methods to produce clinical-grade therapeutic EVs. In addition to loading defined molecules into EVs, engineering their delivery to specific target tissues would be a significant enhancement to such a therapeutic. As such, we have recently developed a platform using membrane cloaking and surface display technology to direct EVs to target tissues. Cloaking lends itself to utilizing any biotinylated antibody – to which countless are commercially available for testing. For example, we previously demonstrated that CDC-EV uptake by cardiac fibroblasts, which is ordinarily minimal, could be augmented *in vitro* by ligating a DDR2 antibody (Antes et al., 2018). Addition of an ischemic targeting peptide to the surface of CDC-EV conferred enhanced *in vivo* targeting to the myocardium. Such an advent may provide significant therapeutic value to target a major cell source contributing to the development of myocardial fibrosis.

## Conclusion

Extracellular vesicles represent a mode of intercellular communication near and far. These tiny vesicles carry messages in the form of biomolecules to inform recipient cells of the current (patho)physiological state and direct the cell to respond appropriately. The recognition that EVs secreted from stem/progenitor cells are therapeutically bioactive when given to animals with heart disease represents a paradigm shift away from cell therapy toward a cell-free platform. Such a paradigm shift overcomes many key concerns and limitations of cell therapy, while conferring the amendable ability to modify vesicle cargo and tissue targeting. Indeed, we and others have demonstrated that payload RNAs, notably miRs, long non-coding RNAs, Y-RNAs, and piRNA, produce transcriptomic and epigenomic modifications that impart lasting effects on recipient cells. By targeting key cellular and molecular players in cardiac fibrosis, these defined molecules contained within therapeutically potent EVs provide insight into avenues for bioengineering. Time will certainly tell if EV-based therapeutics live up to their promise.

## Author Contributions

RR drafted the manuscript. AI and AC generated the figures. EM approved the final version. All authors helped to write the manuscript.

## Conflict of Interest

EM is a founding equity share-holder of Capricor Therapeutics. The remaining authors declare that the research was conducted in the absence of any commercial or financial relationships that could be construed as a potential conflict of interest.

## Footnotes

1 http://www.exocarta.org
